# Quercetin induced NUPR1-dependent autophagic cell death by disturbing reactive oxygen species homeostasis in osteosarcoma cells

**DOI:** 10.3164/jcbn.19-121

**Published:** 2020-03-06

**Authors:** Bowen Wu, Wusi Zeng, Wei Ouyang, Qiang Xu, Jian Chen, Biao Wang, Xiping Zhang

**Affiliations:** 1 Department of Orthopedics, The Affiliated Zhuzhou Hospital Xiangya Medical College CSU, Zhuzhou 412007, China; 2 Department of Oncology, The Affiliated Zhuzhou Hospital Xiangya Medical College CSU, Zhuzhou 412007, China

**Keywords:** quercetin, autophagy, NUPR1, ROS, osteosarcoma

## Abstract

Osteosarcoma is a primary bone aggressive cancer, affecting adolescents worldwide. Quercetin (a natural polyphenolic compound) is a polyphenolic flavonoid compound found in a variety of plants. It has been demonstrated to exert cytostatic activity against a variety of human cancer, including the human osteosarcoma. However, its efficacy in the treatment of osteosarcoma and the underlying antitumor mechanism has not been fully elucidated yet. In this study, we exposed MG-63 cells to different concentrations of quercetin (50, 100 and 200 µM) for 24 h. Here, we show that quercetin increased autophagic flux in the MG-63 cells, as evidenced by the upregulation of LC3B-II/LC3B-I and downregulation of P62/SQSTM1. Moreover, the autophagy inhibitor Bafilomycin A1 or genetic blocking autophagy with ATG5 knockdown decreased quercetin-induced cell death, indicating quercetin triggered autophagic cell death in MG-63 cells. Specifically, quercetin increased NUPR1 expression and activated of NUPR1 reporter activity, which contributed to the expression of autophagy-related genes and subsequent initiated autophagic cell death in osteosarcoma cells. Importantly, the increased expression NUPR1 were tightly related to the disturbance of reactive oxygen species (ROS) homeostasis, which could be prevented by inhibiting intracellular ROS with NAC. Finally, NAC also abolished quercetin-induced autophagic cell death *in vivo*. Taken together, these data demonstrate that quercetin induces osteosarcoma cell death via inducing excessive autophagy, which is mediated through the ROS-NUPR1 pathway. Quercetin application may be a promising and practical strategy for osteosarcoma treatment in clinical practice.

## Introduction

Osteosarcoma (OS) is the most common primary malignant bone tumor, mainly occurring in children and adolescents, accounting for approximately 42% of all primary bone tumors and about 2.4% of all pediatric tumors throughout the world.^(1)^ The combination of surgical resection with intensive chemotherapy have greatly improved the long-term survival rate and clinical remission rate for OS patients, the rate of 5-year overall survival has increased from about 20% to 65–70% for patients with OS.^(2)^ However, there are many side effects and unclear curative effect with the current chemotherapy drugs such as adriamycin, cisplatin, methotrexate, and 5-fluorouracil.^(3)^ Thus, it is urgently needed to research more effective therapeutics and few side effects of drugs to eliminate primary OS and inhibit metastasis to improve the long-term survival rate.

The traditional Chinese medicine (TCM), a very important strategy for the treatment of tumor cells in China, has been a focus for research. Quercetin, chemically known as 3,3',4',5,7-pentahydroxyflavone (C15H10O7), is a bioactive flavonoid that possesses multiple pharmacologic effects such as anti-oxidant, antiaging, anti-inflammatory and antidiabetic activities.^(4,5)^ Remarkably, this compound has recently attracted considerable attention because of accumulating data demonstrating its strong inhibitory effect on various carcinomas, such as lung cancer, hepatocellular carcinoma, and colorectal carcinoma.^(6)^ Also, some studies showed that quercetin can also be used as a pharmacological agent for suppressing the proliferation and inducing the apoptosis of OS cells.^(7,8)^ However, the molecular basis of quercetin is poor defined in OS.

Autophagy is a homeostatic, catabolic degradation process by which the cargo is delivered to the lysosome for degradation.^(9)^ Autophagy has been involved in various anticancer treatments used in clinical today and many therapies that are during the research. It is well accepted that autophagy plays a dual role in cancer development and treatment, as either a tumor suppressor to inhibit tumor progression or a cell survival mechanism to promote tumor growth.^(10)^ On the one hand, autophagy recycles the damaged cellular components to provide substrates for biosynthesis and energy homeostasis.^(11)^ On the other hand, excessive autophagy may lead to overdigestion of the cellular components and eventually to cell death.^(12)^ Thus, the ability of anti-cancer drugs to increase or decrease autophagic flux in human OS cells should be determined.

NUPR1, also known as p8 or Com1, was first described in the pancreatic acinar cells of rats in a study evaluating molecular changes induced by acute pancreatitis.^(13)^ NUPR1 is required for the optimal expression of metabolic stress-response genes, particularly those involved in DNA repair, cell cycle regulation, apoptosis, entosis and autophagy.^(14–16)^ Accumulated evidence has led to the integrating hypothesis that activation of NUPR1 transactivates genes necessary for autophagosome formation, autophagosome-lysosome fusion, and cargo degradation.^(17)^ Importantly, under stress full conditions such as chemotherapy treatment, an intricate interplay between the homeostatic NUPR1 and autophagy pathways may occur in cancer cells that will ultimately dictate their fate between cell death or survival.

In our study, our results demonstrated that quercetin exposure led to induction of autophagic cell death through modulation of the reactive oxygen species (ROS)/NUPR1 pathway in OS cells, and provided experimental evidence to support the future development of quercetin as an effective and safe candidate agent for the prevention and/or therapy of OS.

## Materials and Methods

### Materials

Quercetin (QE), bafilomycin A1 (Baf.A1), DCFH-DA, and *N*-acetyl cysteine (NAC) were purchased from Sigma-Aldrich (St. Louis, MO). NAC was dissolved in distilled water, while the other reagents were dissolved in dimethyl sulfoxide (DMSO), and further dilutions were made with distilled water. DMSO kept at concentrations less than 0.1% had no obvious effect on the cells.

### Cell culture and quercetin treatment

Human osteosarcoma cell line MG-63 cells from the American Type Culture Collection (Manassas, VA) were maintained at 37°C in an atmosphere with 5% CO_2_ with DMEM, supplemented with 10% (v/v) FBS, 100 U/ml penicillin,and 100 µg/ml streptomycin. MG-63 cells were subcultured at 80–90% confluency. The cells used in this study were subjected to no more than 20 cell passages. Quercetin was dissolved in DMSO to obtain a 1 M stock solution and was added directly to the medium at different concentrations of 50, 100 and 200 µM for 24 h.

### Animal experiments

Balb/c nude mice (4 weeks of age) were purchased from Vital River Laboratory Animal Technology Corporation (Beijing, China). They were housed individually in stainless steel cages at a constant temperature (25°C) and a 12-h day/night cycle. The day before the experiment the animals were fasted overnight and were allowed free access to water. The MG-63 cells were trypsinized, counted and re-suspended in sterile PBS. A total of 1 × 10^7^ cells were subcutaneously injected into the left bilateral upper limbs of mice. Investigation of whether NAC co-treatment inhibited quercetin-induced autophagic cell death *in vivo*. The animals were divided randomly into four groups with 10 mice in each group. The first group of mice received normal saline as a control, the second received quercetin (100 mg/kg) treatment alone, the third received NAC (150 mg/kg) treatment alone, and the fourth received NAC and quercetin co-treatment every day for 40 consecutive days. Quercetin, saline and NAC were delivered intragastrically. The tumor volume was measured every 5 days for 40 days. Tumor volume was calculated according to the formula 0.5 × length × width^2^. At the end of the experiment, the tumor tissues were collected for further analysis.

### Cell death assay

MG63 cells were plated in the 6-well plates (5 × 10^5^ cells per well) and incubated for 24 h. After being treated with quercetin, the cells were detached with 300 µl of a trypsin-EDTA solution. The suspension of the detached cells was centrifuged at 300 *g* for 5 min. Then, the pellet was combined with 800 l trypan blue solution and dispersed. After staining for 3 min, the cells were counted using an automated cell counter. The dead cells were stained blue. The cell mortality (%) is expressed as percentage of the dead cell number/the total cell number.^(18)^

### Western blot analyses

Western blot analysis was performed strictly following the method described in our previous research. Tumors were harvested after all mice were sacrificed. The tissue samples were homogenized and sonicated in RIPA buffer on ice. Tissue lysates were then centrifuged at 12,000 *g* for 15 min at 4°C to collect the supernatant. Total cell lysates were extracted with RIPA buffer supplemented with protease inhibitor cocktail following the indicated treatments. The protein obtained from supernatants was quantified according to the standard protocol. Proteins were separated using SDS-PAGE and transferred onto PVDF membranes, which were treated with blocking buffer (5% non-fat dry milk) for 1 h at room temperature before being probed with the following primary antibodies overnight at 4°C, according to the manufacturer’s protocol: rabbit polyclonal anti-NUPR1 (1:1,000, Abcam #ab6028), rabbit polyclonal anti-LC3 (1:1,000, Abcam #ab48394), rabbit monoclonal anti-SQSTM1/P62 (1:1,000, Abcam #ab109012), and rabbit monoclonal anti-ACTB (1:3,000, Abcam#ab115777). The blots were then rinsed and hybridized with corresponding HRP-conjugated anti-rabbit secondary antibodies for 1 h at room temperature. Finally, the membranes were then washed and the visualized using a Luminata Forte Western HRP Substrate and the bands were quantified with ImageJ software.

### RT-PCR

Total RNA was extracted by using TRIzol reagent according to the standard protocol and first-strand cDNA was synthesized using the reverse transcription kit (Takara Biotechnology, Dalian, China). The use of real-time quantitative PCR (RT-PCR) progress and primer sequences was entirely adopted from the previous research. The expression of those genes was calculated using the 2^(–ΔΔ*C*_T_)^ method. The primers used for the amplification of the indicated genes are listed in Table 1.

### Immunofluorescence

Cells were cultured on 10 mm glass-bottom dish (Nest Biotechnology), fixed with 4% paraformaldehyde and permeabilized in 0.1% Triton X-100. After that cells were washed with PBS and blocked with 5% bovine serum albumin (BSA). The cells were incubated with primary antibodies against rabbit polyclonal anti-NUPR1 (1:100, Abcam #ab6028). Coverslips were mounted with the mounting medium (Vector Laboratories) containing diamidino-2-phenylindole (DAPI) and photographed under a laser scanning confocal microscope.

### Luciferase reporter assays

MG63 cells were plated at a density of 1 × 10^4^ cells per well in 96-well culture plates and allowed to reach about 70% confluence. For cotransfection experiments, 0.02 µg of the pcDNA3-NUPR1 expression plasmid or negative control pcDNA3.1 was simultaneously added with reporter plasmids and pRL-TK. Cell lysates were collected at 48 h posttransfection. Firefly and Renilla luciferase activities were measured using the Dual-luciferase Reporter Assay kit (Promega). Firefly luciferase activities were normalized to Renilla luciferase controls.^(19)^

### Transfection

shRNAs were purchased from GeneChem Co., Ltd., Shanghai, China along with control shRNA and Transfection Reagent.^(20)^ MG63 cells were transfected with 100 pM shRNA according to the manufacturer’s protocol. *ATG5*-shRNA: 5'-GAC GUUGGUAACUGACAAATT-3'; *NUPR1*-shRNA: 5'-CTGGTG ACCAAGCTGCAGA-3', and control-shRNA: 5'-TTCTCCGAA CGTGTCACGTTT-3'. Control cells were transfected with a control-shRNA that did not match any known human coding cDNA. At 24 h post-transfection, the cells were exposed to 200 µM quercetin for 24 h.

### Measurement of intracellular ROS

Briefly, the cultured cells were treated with quercetin in the presence or absence of 1 mM NAC for 2 h and subsequently loaded with 0.1 mM DCFH-DA. After incubation for 30 min at 37°C in a 5% CO_2_ incubator, the cells were washed twice with HBSS solution, and examined with an Infinite^TM^ M200 Microplate Reader to detect the intracellular accumulation of ROS.

### MDA assay

Oxidative stress in the tissue samples was measured using a Lipid Peroxidation MDA Assay Kit (Beyotime, Shanghai, China) according to the manufacturer’s directions. MDA levels were detected using an Infinite^TM^ M200 Microplate Reader at 532 nm and are expressed as nmol/mg protein.

### Statistical analysis

Each experiment was performed independently at least three times. The data are expressed as the mean ± SEM. Comparison between groups was performed using One-way ANOVAs. A *p* value <0.05 was considered statistically significant.

## Results

### Quercetin triggers autophagic cell death in MG63 cells

**Fig. 1:**
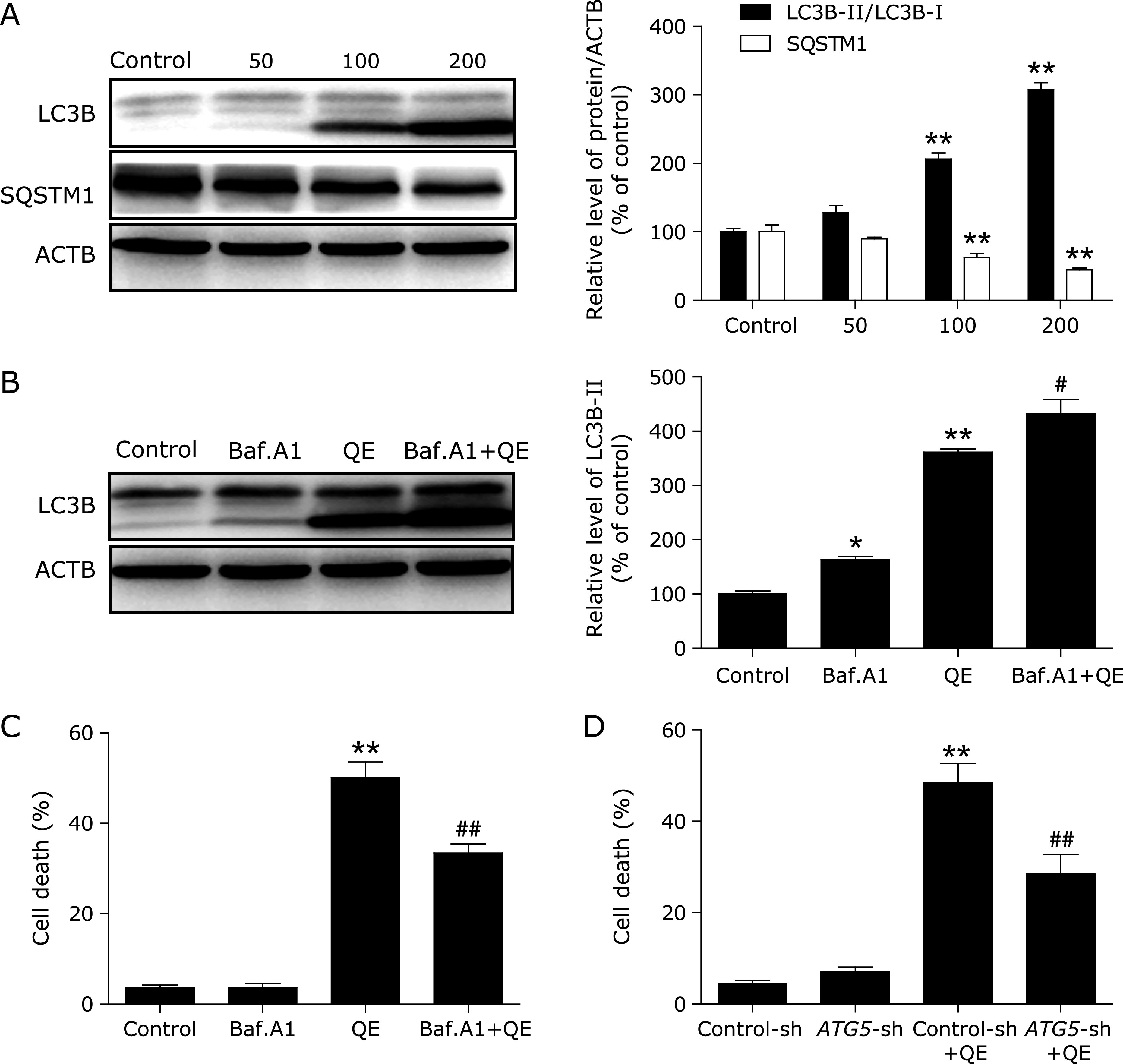
Quercetin induces autophagic cell death in MG63 cells. (A) MG63 cells were treated with different concentrations of quercetin for 24 h, and the expression levels of the autophagy-associated proteins LC3B-I/II, and p62/SQSTM1 were then assessed by western blotting. MG63 cells were pretreated with 100 nM Baf.A1 for 2 h and incubated with 200 µM quercetin for another 24 h. (B) The LC3B-II levels were then assessed by western blotting; MG63 cells were preincubated with (C, D) 100 nM Baf.A1 for 2 h or *ATG5*-shRNA for 24 h and then treated with 200 µM quercetin for another 24 h, then Trypan blue assay used to assess cell death. *p<0.05, **p<0.01 vs the control group, ^#^*p*<0.05, ^##^*p*<0.01 vs the quercetin (200 µM) group (*n* = 4).

To investigate whether quercetin increased or inhibited autophagic flux, we first examined the processing of full-length LC3-I to LC3-II, a hallmark of autophagy, in quercetin-treated MG63 cells. Quercetin increased the protein levels of LC3B-II/LC3B-I ratio in a dose-dependent manner (Fig. 1A). p62/SQSTM1 is selectively incorporated into autophagosomes through direct binding to LC3 and is efficiently degraded by autophagy.^(21)^ We also observed an evident dose-dependent decrease in the p62/SQSTM1 protein levels in MG63 cells that were treated with quercetin, confirming that autophagic flux was intact in the quercetin-treated cell (Fig. 1A). To further confirm autophagy flux is intact after quercetin treatment, we incubated MG63 cells with Baf.A1, which is an inhibitor of the lysosomal V-ATPase and which causes an accumulation of autophagosomes due to a defect in the fusion between autophagosomes and lysosomes,^(22)^ for 2 h prior to exposure to 200 µM quercetin. We found that a Baf.A1 challenge resulted in increased LC3B-II expression in the cells treated with 200 µM quercetin, and these findings demonstrated that quercetin treatment induced autophagic flux in MG63 cells (Fig. 1B). Because the manipulation of autophagy may be involved in the efficacy of anticancer therapeutics, we were eager to assess the effect of quercetin-elicited autophagy on the survival of MG63 cells. As shown in Fig. 1C, Baf.A1 reduced the percentage of cell death from 50.2% to 33.4%. In agreement with the data derived from pharmacological inhibitor, knockdown of ATG5 by shRNA also efficiently protected against quercetin-induced cell death (Fig. 1D). These results show that quercetin induces autophagic cell death in MG63 cells.

### NUPR1 signaling was implicated in autophagy induction by quercetin in MG63 cells

**Fig. 2:**
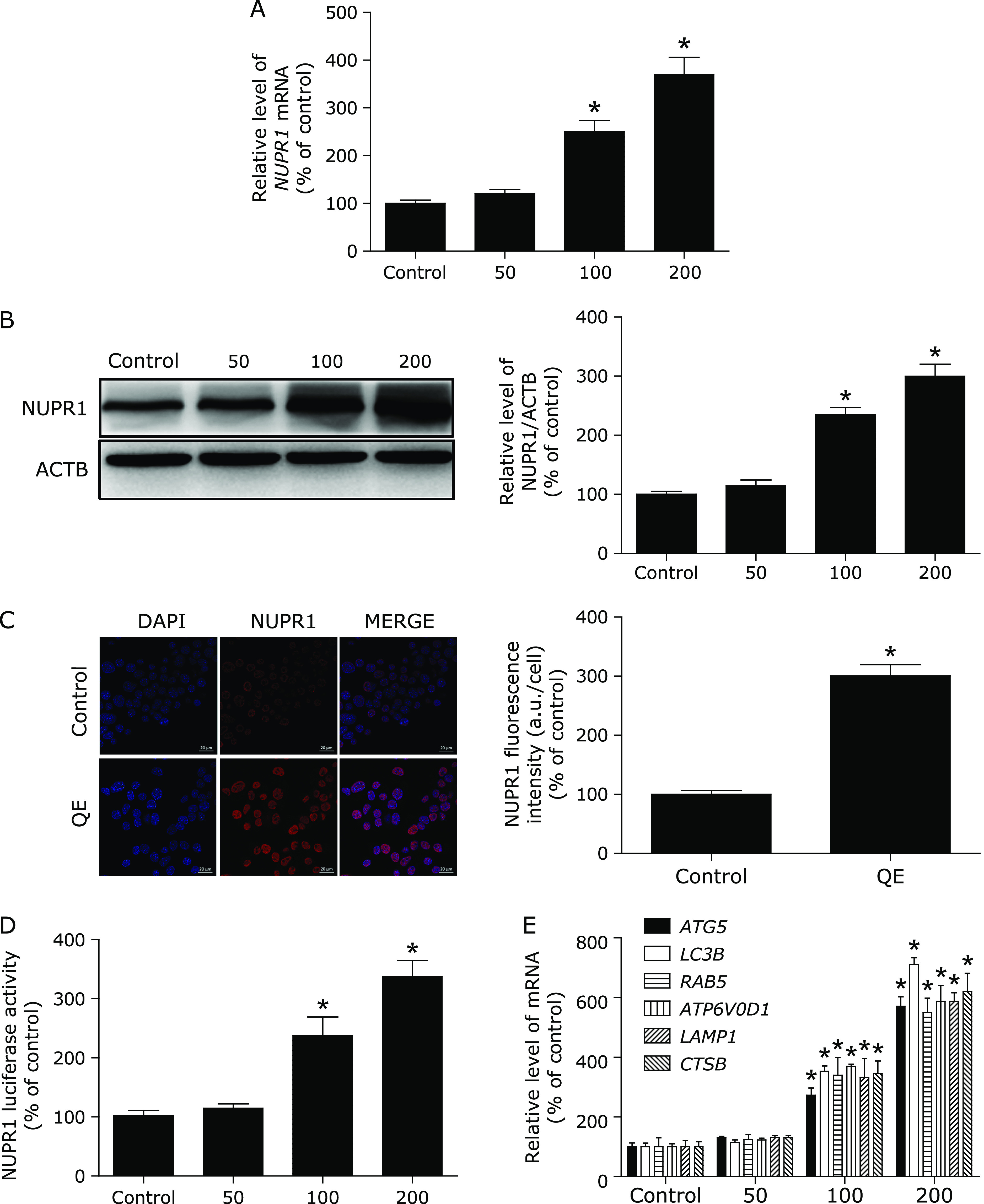
Quercetin increases NUPR1 activity in MG63 cells. (A, B) MG63 cells were treated with different concentrations of quercetin for 24 h, and the mRNA and protein levels of NUPR1 were then analyzed; (C) Immunofluorescence of MG63 cells incubated with anti-NUPR1 antibody and DAPI after quercetin (200 µM) treatment for 24 h; (D) MG63 cells were transfected with NUPR1-luciferase expression vector and incubated with different concentrations of quercetin. The luciferase activity was then measured. (E) The mRNA levels of NUPR1-target genes were measured using RT-PCR. *p<0.01 vs the control group (*n* = 6).

More recently, NUPR1, a master regulator of autophagy flux, has emerged as leading factors in human disease pathology. Based on these results, we investigated whether the NUPR1 is involved in the action of quercetin in MG63 cells. Here, NUPR1 mRNA and protein expression increased significantly after exposure to different concentrations of quercetin for 24 h (Fig. 2A and B). Moreover, the data obtained by immunofluorescence also revealed that NUPR1 was decreased following quercetin exposure (Fig. 2C). As expected, quercetin significantly increased the NUPR1 luciferase activity and the mRNA abundance of 6 tested genes, including ATG5, MAP1LC3B, RAB5, ATP6V0D1, LAMP1, and CTSB (Fig. 2D and E). Taken together, these results indicated an important role for NUPR1 signaling in quercetin-induced autophagy in MG63 cells.

### NUPR1 knockdown inhibits quercetin-induced autophagic cell death in MG63 cells

**Fig. 3:**
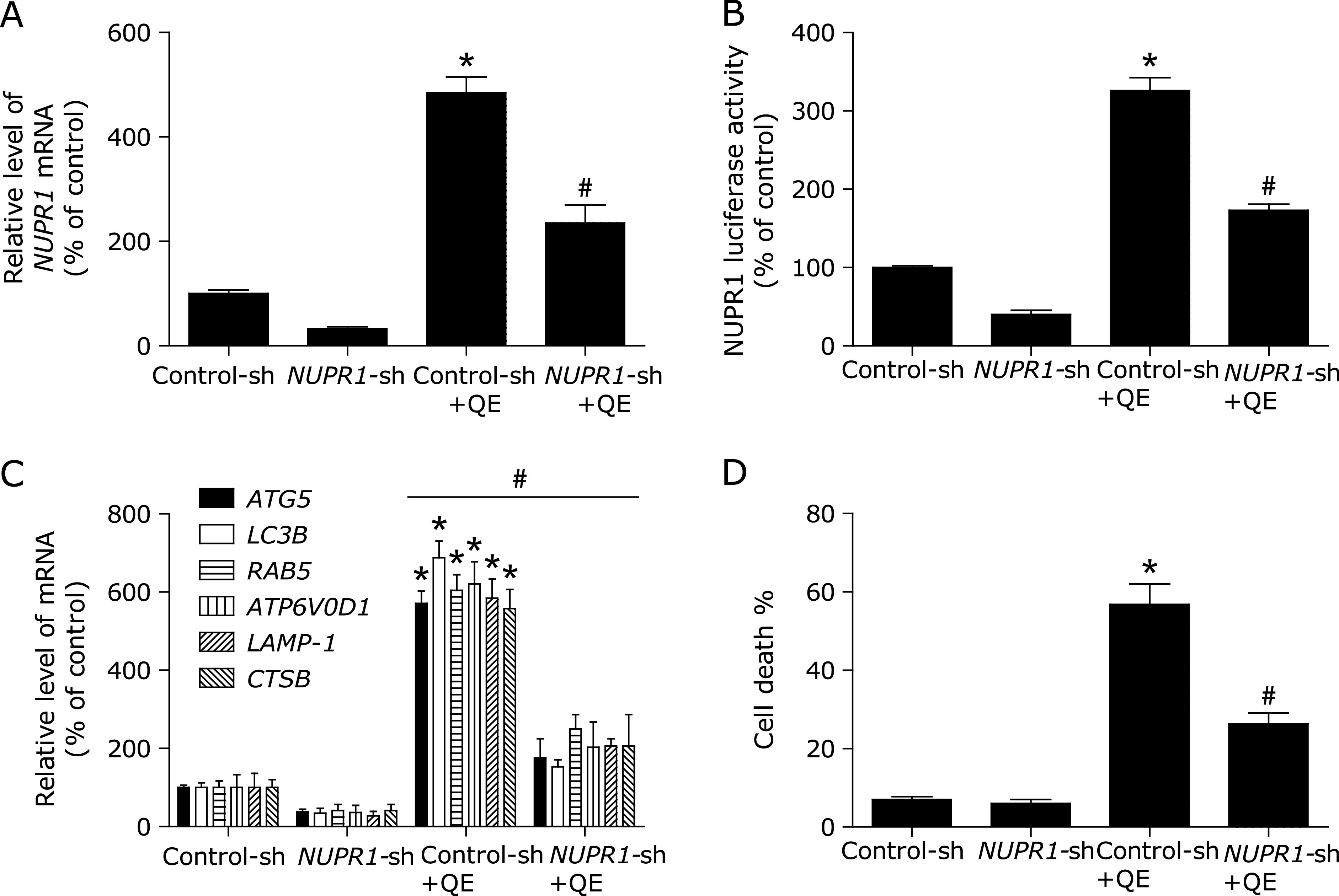
NUPR1 mediates quercetin-induced autophagy. (A) The mRNA levels of *NUPR1* were determined in *NUPR1* shRNA-transfected MG63 cells using RT-PCR. (B, C) MG63 cells were treated with shRNA against *NUPR1* or control shRNA and then incubated with 200 µM quercetin for another 24 h before measuring the Luciferase activity and NUPR1-target genes. (D) Trypan blue assay used to assess cell death. *p<0.01 vs the control group, ^#^*p*<0.01 vs the Control-sh+quercetin (200 µM) group (*n* = 4).

To further confirm the molecular mechanism underlying modulation of autophagy by NUPR1 transcriptional factor in quercetin-treated MG63 cells, we examined the effect of NUPR1 gene silencing on quercetin-induced autophagy in MG63 cells. As shown in Fig. 3A and B, NUPR1-shRNA abrogated quercetin-induced the NUPR1 mRNA levels and luciferase activity. Notably, the inhibition of NUPR1 activity decreased quercetin-induced autophagy-related genes expression (Fig. 3C). Additionally, the quercetin-induced MG63 cell death were also suppressed (Fig. 3D). These results suggested that quercetin induced autophagic cell death by activating NUPR1 pathway in MG63 cells.

### ROS is an upstream signaling molecule that activates the NUPR1-dependent autophagy pathway

**Fig. 4:**
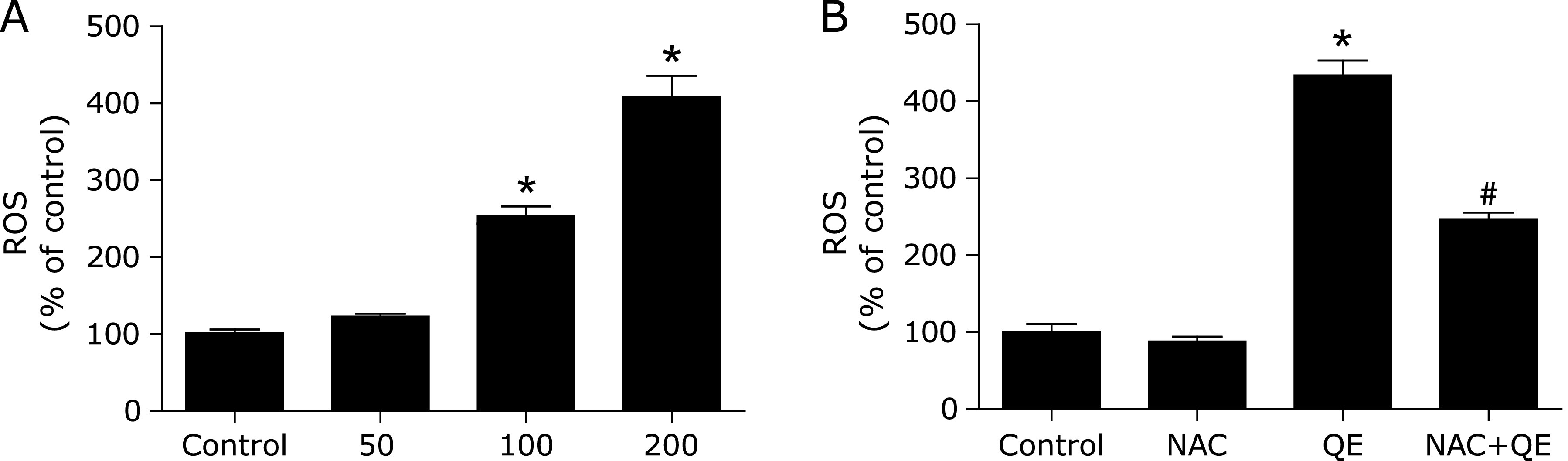
Quercetin increases ROS levels in MG63 cells. (A) The ROS levels were measured with an Infinite^TM^ M200 Microplate reader; MG63 cells were exposed to NAC (1 mM) for 2 h, followed by treatment with quercetin (200 µM) for 24 h. (B) The ROS levels were measured with an Infinite^TM^ M200 Microplate reader; *p<0.01 vs the control group; ^#^*p*<0.01 vs the quercetin (200 µM) group (*n* = 4).

**Fig. 5:**
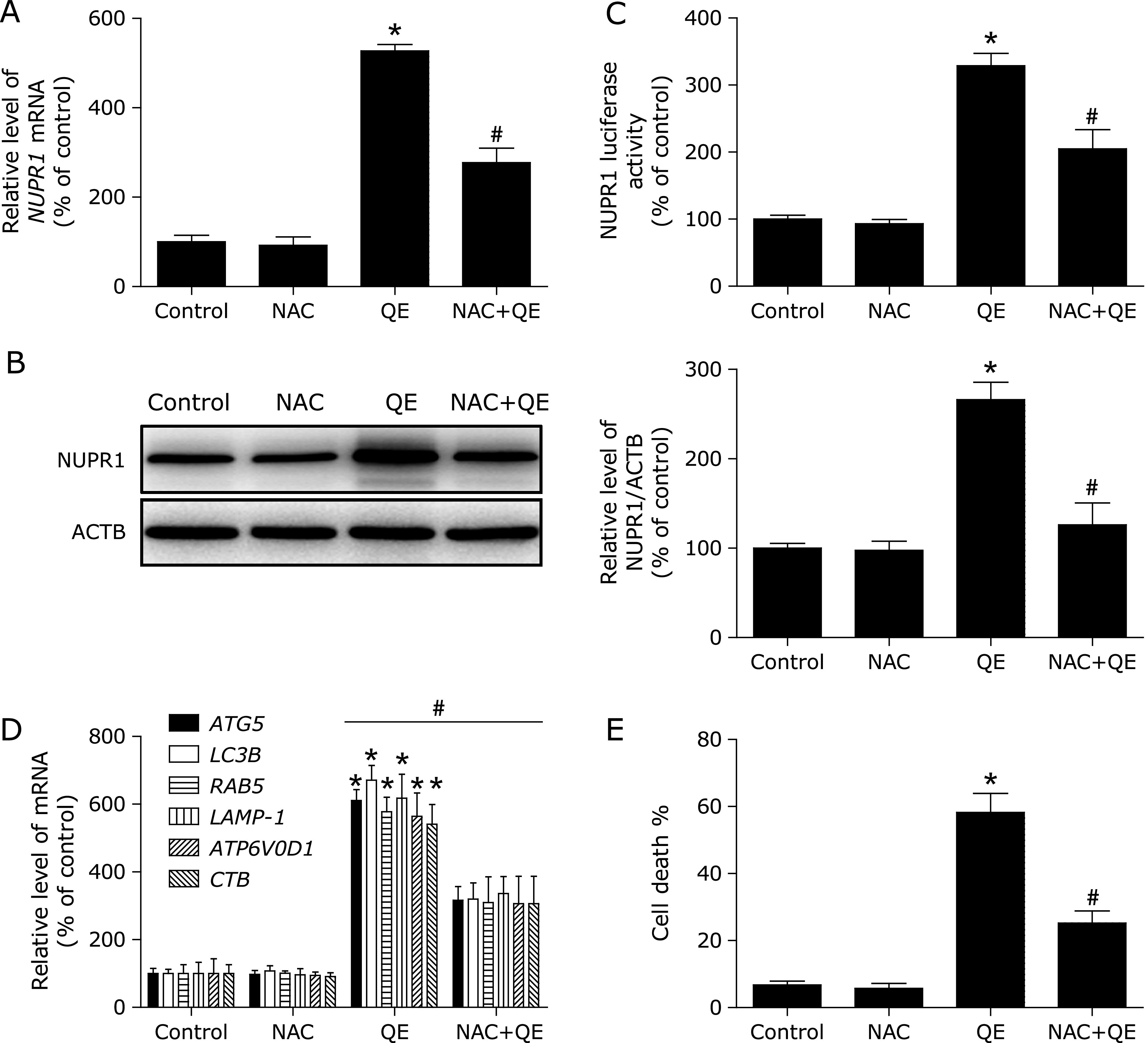
ROS is an upstream signaling molecule that activates the NUPR1-dependent autophagy pathway. (A, B) The mRNA and protein levels of NUPR1 were detected. (C) The NUPR1 Luciferase activity was then measured. (D) The mRNA levels of NUPR1-target genes were measured using RT-PCR. (E) Trypan blue assay used to assess cell death. *p<0.01 vs the control group; ^#^*p*<0.01 vs the quercetin (200 µM) group (*n* = 4).

For ROS homeostasis is closely associated with NUPR1 expression, we finally monitored the concentrations of ROS in quercetin-treated MG63 cells using the ROS indicator DCFH-DA. As shown in Fig. 4A, ROS was significantly increased in a dose-dependent manner. In addition, pre-treatment with specific ROS inhibitor, NAC, decreased ROS levels, inhibited NUPR1 expression, prevented NUPR1 transcription activity, and suppressed autophagy-related genes expression and reduced cell death (Fig. 4B and Fig. 5).

### NAC inhibited quercetin-induced NUPR1-depended autophagic cell death *in vivo*

**Fig. 6:**
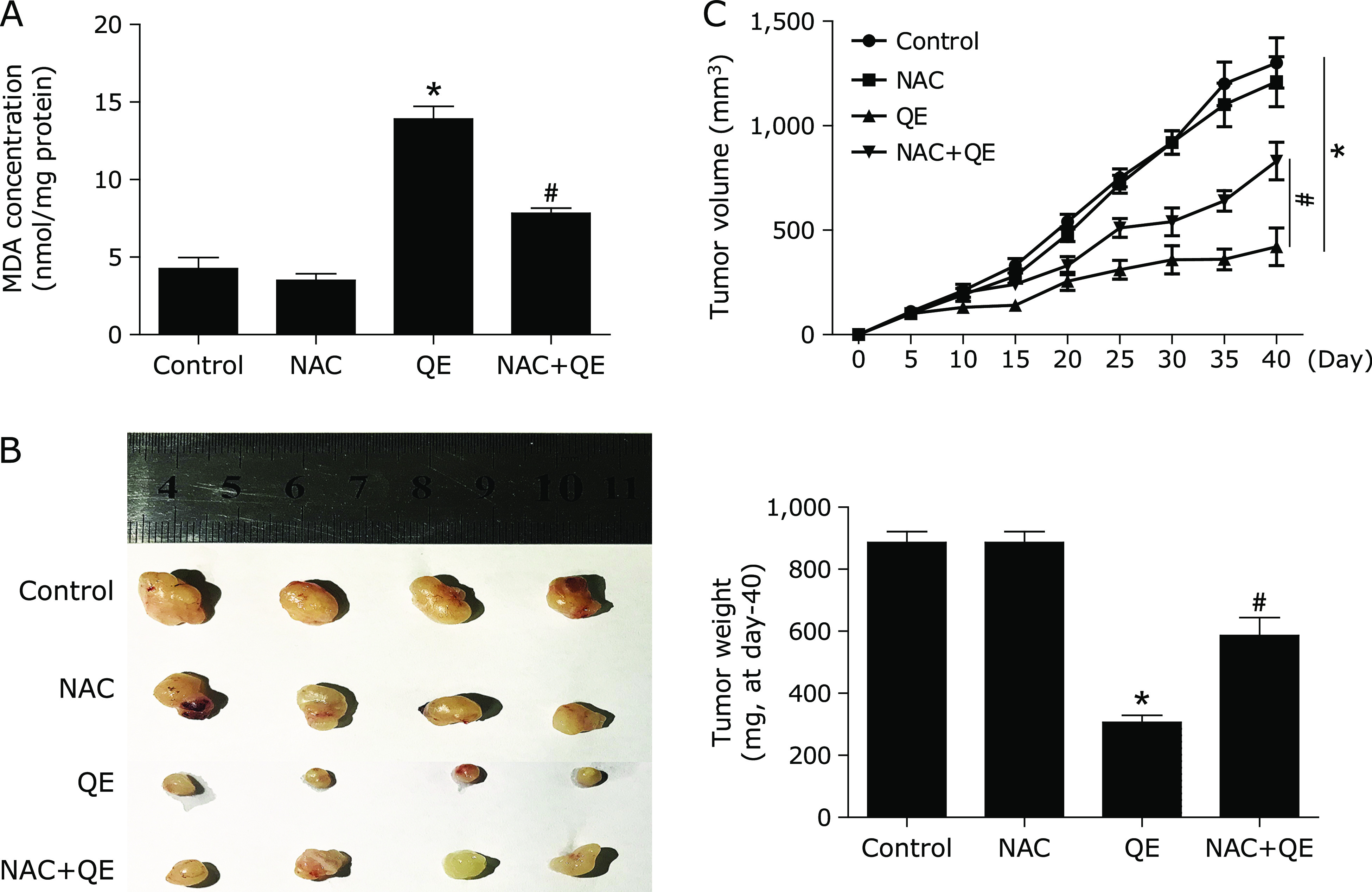
NAC abolishes anti-tumor effect of quercetin *in vivo*. Nude mice with MG63 cells subcutaneous tumor xenografts were established and treated with vehicle control, 100 mg/kg quercetin, with or without 150 mg/kg NAC. (A) The MDA level was determined. (B, C) After excision from the mice, the xenografts were photographed, and the tumor volume and weight were measured. *p<0.01 vs the control group; ^#^*p*<0.01 vs the quercetin group (*n* = 8).

**Fig. 7:**
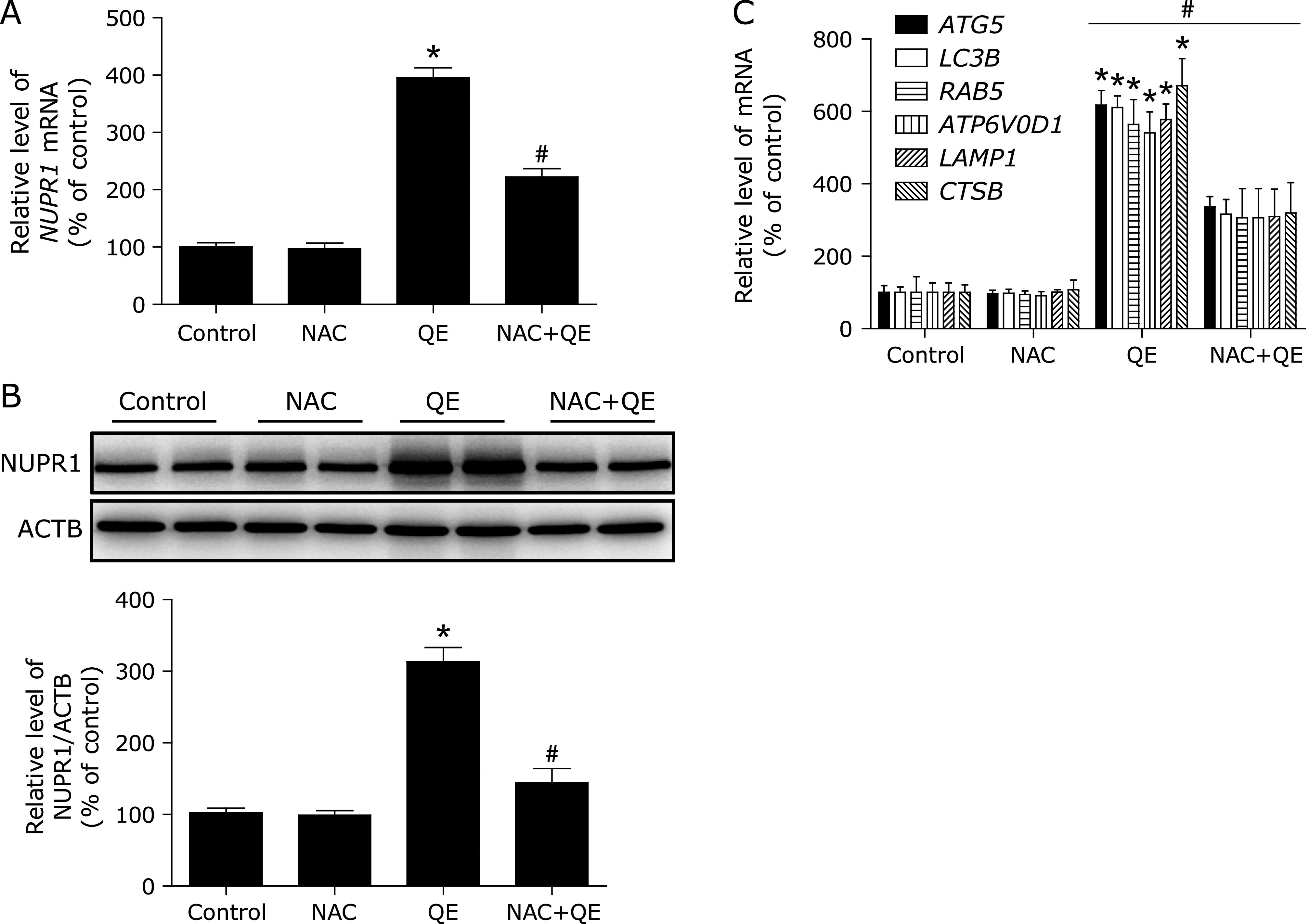
NAC abolishes quercetin-induced NUPR1-depended autophagic cell death *in vivo*. Nude mice with human MG63 cells subcutaneous tumor xenografts were established and treated with vehicle control, 100 mg/kg quercetin, with or without 150 mg/kg NAC. (A, B) The mRNA and protein levels of NUPR1 were detected. (C) The mRNA levels of NUPR1-target genes were measured using RT-PCR. *p<0.01 vs the control group; ^#^*p*<0.01 vs the quercetin group (*n* = 8).

Based on the *in vitro* findings, we investigated whether the NAC could suppress the antitumor effect of quercetin *in vivo*. A xenograft tumor model was established by the subcutaneous inoculation of MG63 cells into nude mice. As shown in Fig. 6A, MDA level was significantly increased in after quercetin treatment, and pre-treatment with specific ROS inhibitor, NAC, decreased MDA levels. Moreover, the antitumor ability of quercetin combined with NAC was lower than that of quercetin alone (Fig. 6B and C); Furthermore, pre-treatment with NAC decreased NUPR1 expression, suppressed autophagy-related genes expression (Fig. 7). Taken together, NAC also abolished quercetin-induced NUPR1-depended autophagic cell death *in vivo*.

## Discussion

The present study was undertaken to shed more light on the mechanisms by which quercetin exerts its anti-cancer activity in cultured OS cells. For the first time, we have demonstrated that (i) quercetin induced autophagic cell death in MG63 cells; (ii) quercetin increased NUPR1 expression and activated NUPR1 reporter activity, which contributed to the expression of autophagy-related genes; and (iii) quercetin increased NUPR1-dependent autophagic cell death by disturbing ROS homeostasis. Taken together, these findings should increase the current understanding of the mechanism of OS treatment of quercetin.

The role of autophagy in cancer chemotherapy with quercetin is still controversial. Cancer cells may utilize autophagy to survive in the hostile tumor microenvironment, suggesting deployment of therapeutic strategies to block autophagy for cancer therapy. For example, quercetin induced cytoprotective autophagy in HL-60 cells, and inhibition of autophagy may be a novel strategy to enhance the anticancer activity of quercetin in AML.^(23)^ Moreover, quercetin also induces protective autophagy in ovarian cancer,^(24)^ lymphoma^(25)^ and glioma.^(26)^ However, high levels of autophagy might lead to cell death in cancers. Jia *et al.*^(27)^ reported that quercetin suppresses the mobility of breast cancer by suppressing glycolysis through autophagy induction. In addition, quercetin inhibits growth of hepatocellular carcinoma by apoptosis induction in part via autophagy stimulation in mice.^(28)^ Consistent with those previous studies, our results revealed that, autophagy inhibitor Baf.A1 or *ATG5*-shRNA could avert the effect of quercetin on cell death, which indicated that quercetin-induced autophagic cell death in OS cells.

The transcription factor NUPR1 acts as a master regulator of cellular clearance through enhancement of the autophagy-lysosome pathway, including expression of autophagy genes, lysosomal biogenesis and lysosomal proteostasis. The function of NUPR1-depened autophagy contributing to cell death is still controversial, with both beneficial and detrimental roles suggested. Recently, Hamidi *et al.*^(17)^ found that NUPR1 is involved in a defense mechanism that promotes pancreatic cancer cell survival when exposed to metabolic stress, and Matsunaga *et al.*^(29)^ reported that deferoxamine-induced NUPR1 promoted mesenchymal stem cell survival and cytoprotective autophagy. Moreover, NUPR1 maintains autolysosomal efflux and is required for the progression of non-small cell lung cancer.^(15)^ However, Tang *et al.*^(30)^ have shown that NUPR1 is activated, and mediates intestinal epithelial cells death via increasing autophagy-related gene in response to Shiga toxins toxicity. Consistent with previous reports, the present study demonstrated that quercetin induced NUPR1-dependent autophagic cell death in OS cells. Importantly, this study now joins several recent studies that have unexpectedly demonstrated favourable results of NUPR1 activation in cancer chemotherapy.

The accumulation of ROS to NUPR1 inhibition has been reported in recent studies. The Sebastian Weis group reported that NUPR1 deficiency increases cellular ROS in mouse embryonic fibroblasts.^(31)^ Moreover, NUPR1 downregulation induces mitochondrial failure with loss of the mitochondrial membrane potential, a strong increase in ROS production and promotion of pancreatic cancer cell death.^(16)^ In the present study, quercetin exposure was found to initiate ROS elevation, and inhibiting ROS efficiently decreased NUPR1 expression, followed by a reduction of autophagy-related genes expression. Our result is contrary to previous findings. We propose there is a loop between ROS and NUPR1 for this phenomenon, and the details of the mechanisms require further research.

Great attention has been paid to the anti-tumor effects of NAC. Recently, Monti *et al.*^(32)^ reported that NAC as a single agent reduced MCT4 stromal expression, which is a marker of glycolysis in breast cancer with reduced carcinoma cell proliferation in a clinical trial. However, other previous studies confirmed NAC inhibited anti-tumor effect of some chemical agents isolated from the plant, such as britannin,^(33)^ Umbilicaria esculenta,^(34)^ and cryptotanshinone.^(35)^ Consistent with those previous studies, NAC also abolished quercetin-induced NUPR1-depended autophagic cell death in OS cells both *in vitro* and *in vivo*. These results also suggested that clinical trials will need to be performed to test if NAC combined with other antioxidants or metabolic modulators are effective in OS.

In summary, quercetin induces NUPR1-dependent autophagic cell death by disturbing ROS homeostasis in MG63 cells. Thus, these compelling evidences indicated that quercetin may be a potential and effective candidate against human OS for its well anticancer efficiency and high safety.

## Figures and Tables

**Table 1:** Sequences of primers used in quantitative RT-PCR

Gene name		Nucleotide sequence
*ATG5*	F	5'-ACTCCAATCCTGTGAGGCAG-3'
	R	5'-TCCTCTGGCATTCACACCG-3'
*ATP6V0D1*	F	5'-TTCCCGGAGCTTTACTTTAACG-3'
	R	5'-CAAGTCCTCTAGCGTCTCGC-3'
*CTSB*	F	5'-GAGCTGGTCAACTATGTCAACA-3'
	R	5'-GCTCATGTCCACGTTGTAGAAGT-3'
*RAB5*	F	5'-GCTAATCGAGGAGCAACAAGAC-3'
	R	5'-CCAGGCTTGATTTGCCAACAG-3'
*LAMP-1*	F	5'-TCTCAGTGAACTACGACACCA-3'
	R	5'-AGTGTATGTCCTCTTCCAAAAGC-3'
*LC3B*	F	5'-AGCAGCATCCAACCAAAATC-3'
	R	5'-CTGTGTCCGTTCACCAACAG-3'
*NUPR1*	F	5'-GCACGAGAGGAAACTGGTGA-3'
	R	5'-GTCCCGTCTCTATTGCTGGG-3'
*ACTB*	F	5'-CATGTACGTTGCTATCCAGGC-3'
	R	5'-CTCCTTAATGTCACGCACGAT-3'
